# Acute outcomes of acupuncture and dry needling over peripheral acute fatigue in untrained healthy volunteers: A randomized controlled clinical trial

**DOI:** 10.1016/j.heliyon.2023.e20093

**Published:** 2023-09-12

**Authors:** Gabriel Antonino, Ana Paula Ferreira, Horianna Mendonça, Lívia Shirahige, Eduardo Montenegro, Marcelo Guerino, Alberto Filho, Mario Bernardo-Filho, Shirley Lima Campos, Wagner Souza Leite, Kátia Monte-Silva, Redha Taiar, Amandine Rapin, Maria das Graças Rodrigues de Araújo

**Affiliations:** a Laboratory of Applied Neuroscience, Universidade Federal de Pernambuco, Recife, Brazil; b Laboratory of Kinesiotherapy and Manual Therapy Resources, Universidade Federal de Pernambuco, Recife, Brazil; c Laboratory of Electrotherapy and Thermotherapy, Universidade Federal de Pernambuco, Recife, Brazil; d Mechanical Vibration Laboratory and Integrative Practices, Universidade Do Estado Do Rio de Janeiro, Rio de Janeiro, Brazil; e Multiuser Laboratory of Instrumental Innovation and Physical Performance, Universidade Federal de Pernambuco, Recife, Brazil; f MATériaux et Ingénierie Mécanique (MATIM), Université de Reims Champagne-Ardenne, Reims, France; g CHU de Reims, Hôpital Sébastopol, Service de Médecine Physique et de Réadaptation, 51092, REIMS, France; h Université de Reims Champagne Ardenne, Faculté de Médecine, UR 3797 VieFra, 51097, REIMS, France

**Keywords:** Muscle fatigue, Acupuncture, Dry needling

## Abstract

Peripheral acute fatigue (PAF) is defined as when the skeletal muscle is incapable of generating power. We aimed to investigate the acute effects of traditional Chinese acupuncture (TCA) and dry needling (DN) over PAF induced on the biceps brachii of untrained healthy volunteers. We conducted a randomized, single-blind controlled clinical trial. All volunteers (n = 45) underwent fatigue induction protocols repeated before and after treatment with TCA (TCA group; TCAg; n = 15), DN (DN group; DNg; n = 15), and rest (control group; Cg; n = 15). Assessments of PAF, skin temperature, and exercise time occur before and after each event: 1st fatigue induction (FI), treatment, and 2nd FI. We used repeated measures ANOVA adjusted with Bonferroni post hoc test to determine any change in tested variables (PAF-VAS, PAF-EMG, and skin temperature) at different time points compared to the baseline. Paired Samples *t*-test was used for the variable exercise times. All statistical tests considered’ the significance level at p ≤ 0,05. There was no difference between groups in acute fatigue recovery (p = 0.19). All intragroup analyses were significant (p ≤ 0.05) and all volunteers show a reduction in fatigue perception after treatment (p ≤ 0,05), however, exercise time did not ameliorate after TCA or DN (p > 0.77). A single session of TCA and, DN can equally reduce fatigue, temperature, and exercise time over PAF induced on biceps brachii of untrained healthy volunteers.

## Simple summary

Some therapies might reduce muscle fatigue; however, we are yet to know how traditional Chinese acupuncture and dry needling might affect muscle fatigue, therefore, we aimed to investigate the acute effects of traditional Chinese acupuncture and dry needling over peripheral acute fatigue induced on biceps brachii of untrained healthy volunteers through a single-blind controlled clinical trial. We accessed muscle fatigue itself with electromyography and fatigue perception, we also accessed skin temperature and exercise time. Even though our results show that there was no difference between treatments on fatigue recovery, all volunteers show a reduction in fatigue and fatigue perception. There was a temperature reduction after both treatments and exercise time did not have any alterations. Our results might help to better understand how acupuncture and dry needling might affect induced fatigue and guide other researchers to perform other studies to elucidate mid- and long-term effects not only in healthy untrained men but also, in disease-related populations.

## 1 Introduction

Peripheral acute fatigue (PAF) is when skeletal muscle is incapable to generate power or strength after exercise or repetitive and extenuating activities, as well as the capability to maintain optimal performance and achieve maximum voluntary contractions [1,2] leading us to understand that PAF is tethered to a muscle effort [2] and human performance [3]. Muscle performances promote micro-lesions of muscle fibers generating an inflammatory process [4] first with rapid autonomic reflex vasoconstriction followed by vasodilation inducing blood flow increase directly related to muscle activity [5]. A state of peripheral fatigue overlaid with the inflammatory process might last for days, depending on what muscles were evoked, negatively influencing any given task with social and economic proportions [6,7]. Previous studies found that fatigue reduction might improve not only daily living tasks but also, diseases and sports recovery [8,9]. Therefore, we found that investigating innovative therapies for fatigue recovery is of utmost importance to promote faster and inexpensive fatigue recovery.

Over the past decade, several acupuncture studies emerged to elucidate its effects on an occidental optic [5,[10], [11], [12]]. Traditional Chinese acupuncture (TCA) uses needles inserted in specifics points (acupoints) through 14 main meridians in the human body [13] used frequently to treat many human conditions including chronic and acute pain [12], hypertension [11], Parkinson's [14], and fatigue itself, both peripheral [15] and chronic [10] occurring through excitatory and inhibitory effects in the motor system and improve function [16] along with vasoactive effects of TCA in the human body have also been studied regarding reverberations over skin temperature [5,17]. However, we are yet to understand how TCA might affect immediate muscle recovery after induced peripheral fatigue.

Alongside TCA, dry needling (DN) therapy has been the focus of many studies and, as a needling therapy, promotes similar effects regarding vasoactive [18,19] and skin temperature changes [20,21], pain relief [[22], [23], [24]] and fatigue recovery [25,26]. There are, indeed, many similarities between TCA and DN [27], although, we can differentiate DN from TCA through their theory, needling insertion variations, and sites [28]. Nevertheless, our understanding of the impact of TCA and DN on muscle fatigue, skin temperature, and exercise duration following peripheral fatigue induction remains uncertain.

In addition, with the increase in muscular activity, there is an inevitable rise in cutaneous temperature due to vasoactive changes [29], thus, it is possible to access PAF with body temperature, such as the infrared thermographic imaging (ITI) [30,31]. ITI is an evolving technology within various research fields as it can assess skin temperature in any condition that alters thermoregulatory mechanisms [20,31], especially in breast cancer [32] joint [33,34], and skeletal muscle lesions [35] in which we can predict with ITI assessment as well as an indication of PAF [30,36].

Fatigue is unique for each individual including its perception, which leads us to understand it as a psychophysiological factor generated by personal subjective sensation related to any energy expenditure associated with muscle activity [37]. Perception of effort is widely applied in fatigue assessments, allowing us to use a visual analog scale (VAS) to assess FAP perception related to exercise and recovery [38,39].

Many studies have shown positive effects on fatigue recovery using rest [40], cryotherapy [41], electrotherapy [42], and non-invasive brain stimulation [38]. Another treatment that has been studied for fatigue recovery is traditional Chinese acupuncture (TCA) with long-lasting physiological effects [15].

To our best knowledge, there are no studies that compare TCA and DN acute effects of biceps brachii peripheral fatigue over fatigue, skin temperature, and exercise time. Given the need to identify rapid, low-cost, and effective treatments for fatigue recovery and describe how TCA and DN immediate effects alter PAF, skin temperature, and exercise time, the present study aimed to investigate the acute effects of TCA and DN over PAF induced on biceps brachii of untrained healthy volunteers.

## 2 Materials and methods

### 2.1 Study design

We conducted a three-arm single-blinded randomized controlled trial enabling direct treatment comparisons while minimizing bias. We recruited volunteers between February 26, 2018, and December 21, 2018. Forty-five volunteers were randomized into three groups: the traditional Chinese acupuncture group (TCAg; n = 15), the dry needling group (DNg; n = 15), control group (Cg; n = 15). All treatments and assessments were carried out at the *Laboratório de Cinesioterapia e Recursos Terapêuticos Manuais* (Laboratory of Kinesiotherapy and Therapeutic Manual Recourses) at the *Universidade Federal de Pernambuco* (Federal University of Pernambuco) after the Research Ethics Committee in Human Beings approval under no. 2.419.094 and registered at Clinical Trials under no. NCT03448120**,** with the first entry on February 21, 2018. The volunteers were randomly allocated through blocked randomization into the three groups using the website www.randomization.com, following the CONSORT guidelines (see CONSORT flow diagram in Fig. 2). All volunteers signed an informed consent document before participating as foreseen by Resolution 466/12 of the National Health Council. The study was performed according to the principles of the Declaration of Helsinki.

### 2.2 Sample

A priori sample size was calculated using G*Power 3.1 with data from Toma et al.*,* [43] based on an expected strength performance difference of 0.42 kg/F, α = 0.05, and statistical power of 90% resulting in a sample of 34 subjects.

The sample of this study consisted of 45 healthy male individuals, who did not practice physical exercise, randomly chosen among students and employees of UFPE through a database with 71 volunteers who were interviewed, aged between 18 and 40 years old, and who responded to the International Physical Activity Questionnaire (IPAQ) short form validated to Brazilian Portuguese [44].

We excluded volunteers who were using food supplementation, presented lesions or bruises in the studied upper limb, had metabolic problems, were in hormone therapy, and ingested caffeine within 2 h before the study. In addition, who presented myofascial trigger points in the areas of needle insertions, BMI below 18.5 or above 29.9, and “active” or “very active” volunteers, according to the IPAQ short version.

### 2.3 Outcome measures

#### 2.3.1 Peripheral acute fatigue

The volunteer underwent a protocol of a series of isometric contractions to induce PAF with a load cell (SDS 1000 S rigid load cell of the Miotec®) attached to surface electromyography (Electromyograph Miotool SDS 500, from MIOTEC®, with 4.5 × 3.8 cm disposable Ag/AgCl surface electrodes). We used Surface Electromyography for Non-Invasive Assessment of Muscle (SENIAM) guidelines [45] for electrode placement and electromyography (EMG) assessment of the biceps brachii.

As described in a previously published protocol [46], the volunteer performed a maximum voluntary isometric contraction (MVIC) followed by a 10-min rest for acclimatization. Both MVIC and fatigue induction protocols were performed in a laid position on a stretcher with movable arms. The volunteer's non-dominant arm was positioned with a 90° shoulder abduction, along with the stretcher arm, 90° elbow flexion and, full supinated forearm and close fist. A strap attached to the volunteer's wrist was connected to a load cell attached to a wall to measure force together with the EMG assessment.

**Fig. 1:**
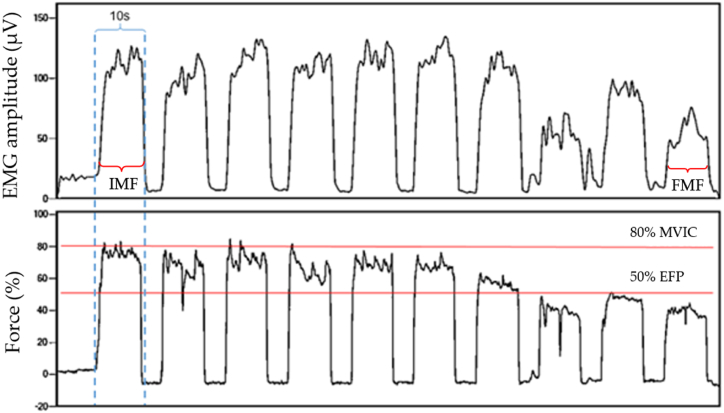
Intermittent contractions in EMG (μV), Force (percentage of kgF), and exercise time data acquisition. IMF: initial median frequency; FMF: final median frequency; MVIC: maximum voluntary isometric contraction; EFP: exercise failure point.

**Fig. 2:**
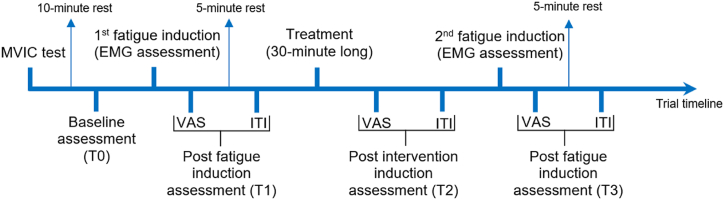
All assessments consisted of EMG, VAS, ITI, and exercise time. EMG assessment consisted of median frequency analysis in addition to exercise time (fatigue induction). The trial consisted of one single session that lasted for 60 min. The applied treatment varied according to the group allocation (TCAg, DNg, or Cg).

We performed intermittent contractions of 10 s with 5 s resting periods with no generated force using 80% of the MVIC for the exercise load and 50% associated with a homogeneous fall of muscle force periodogram as an exercise failure point (EFP) in two distinct moments (1st and 2nd fatigue induction; Fig. 2) which the volunteer ceased contractions as reached EFP as seen on Fig. 1. PAF was indicated with median frequency analysis of EMG of the mean of the first and last contractions, resulting, respectively, in the initial median frequency (IMF; T0) and final median frequency (FMF; T1). We analyzed PAF recovery comparing T1 with IMF of the 2nd fatigue induction (T2).

#### 2.3.2 Peripheral acute fatigue perception

A 10 cm scale with a “no fatigue” field (0) at one end and “maximum fatigue” (10) on the opposite end was used. Participants made a mark along the scale to identify their level of fatigue. We considered PAF only for the biceps brachii at baseline (T0), immediately after: 1st fatigue induction (T1), intervention (T2), and 2nd fatigue induction (T3).

#### 2.3.3 Muscle temperature

We used a non-cooled thermographic camera (FLIR® Model E40bx), with an emissivity of 0.98, to monitor the temperature of the human body surface. Its lens was directed to the Region of Interest (RI) attached on an adapted tripod; one should expect an average of 10 min for the uncooled camera to adjust to the ambient conditions (temperature and relative air humidity) and electronic devices in the same environment, that is, acclimatization [47,48].

The ITI data assessment room had a minimum of 6–9 m^2^, with black walls, and its temperature was between 18 and 25 °C, as well as the relative air humidity, which was between 40 and 70% [47]. The parameters of ambient temperature and relative humidity were constantly monitored with an indoor thermo-hygrometer, model TA298.

The temperature variable used for ITI analysis is the mean temperature (MT) of the RI adapted from the Glamorgan protocol [48], in an ellipsoid shape appropriate to the area of the medial view of the BB in which its vertices were aligned with the axillary line and the cubital fossa of the volunteer. The ITI was captured in four moments: Baseline (T0), 5 min after the first fatigue induction (T1), immediately after each treatment (T2), and 5 min after the second fatigue induction (T3) (Fig. 2).

#### 2.3.4 Exercise time

The exercise time was the intermittent isometric contractions delimited by the number of valid contractions during each fatigue induction: 1st (T1) and 2nd (T2). A valid contraction equals 10 s of muscle activity for each isometric contraction. Rest intervals during exercise were not measured (Fig. 1).

### 2.4 Treatments

The TCA group (TCAg) underwent needles application in the following acupoints in this order: Large intestine (LI1; Hegu), Triple heat (TH5; Waiguan), LI10 (Shousanli), LI11 (Quchi), LI14 (Binao) e Gallbladder (GB21; Jianjing) [49].

To localize each of the six acupoints we used the *tsun* measure, which corresponds to the volunteer's interphalangeal or thumb measurements. This measure is unique, therefore, each new volunteer was assessed with a manual caliper. The needle direction was at 90° concerning the cutaneous surface. Its depth varies with the topographic site of each acupoint [50].

Same as TCAg, in the DN group (DNg) six needles were inserted into the volunteer's bicipital region so that biases related to dosimetry are prevented. The application of the needles followed this arrangement: needle one (center of the muscle belly); needle two (2 cm below needle one) needle three (2 cm above needle one); needle four (biceps long head tendon); needle five (biceps insertion tendon) and needle six (bicipital retinaculum). To apply the TCA and DN, we used disposable filiform needles from DongBang® with 0.25x40mm [25,51]. The volunteers rested for the same time as the subjects from the TCAg e DNg underwent the therapies [15,52].

### 2.5 Trial timeline

All assessments and outcomes are presented in Fig. 2.

### 2.6 Randomization and blinding

The study was performed by ten independent researchers: (1) one researcher was responsible for the recruitment, application of questionnaires, anthropometric data, randomization, and VAS assessment; (2) another researcher acquired and processed the ITIs, as well as application of the DN; (4) an experienced and previously trained researcher performed the TCA; (5) a researcher performed exclusively the statistical analysis; (6 and 7) research that revised the manuscript; (8 and 9) researchers were entitled to a critical review of the paper. Researcher 5 and the volunteers were blind to all treatments.

### 2.7 Statistical analysis

Data were analyzed using SPSS 23.0 (IBM Corp, Armonk, NY), and were presented as means and standard deviation (SD) or 95% confidence interval (95%CI). Normal distribution was assessed with the Shapiro-Wilk test. Comparisons among TCA, DN, or C groups were conducted by using repeated measures ANOVA adjusted with Bonferroni post hoc test to determine any change in tested variables (PAF-VAS, PAF-EMG, and skin temperature) after at different time points compared to baseline. Paired Samples *t*-test was used for the variable exercise times. All statistical tests considered the significance level at p ≤ 0.05.

## 3 Results

**Fig. 3:**
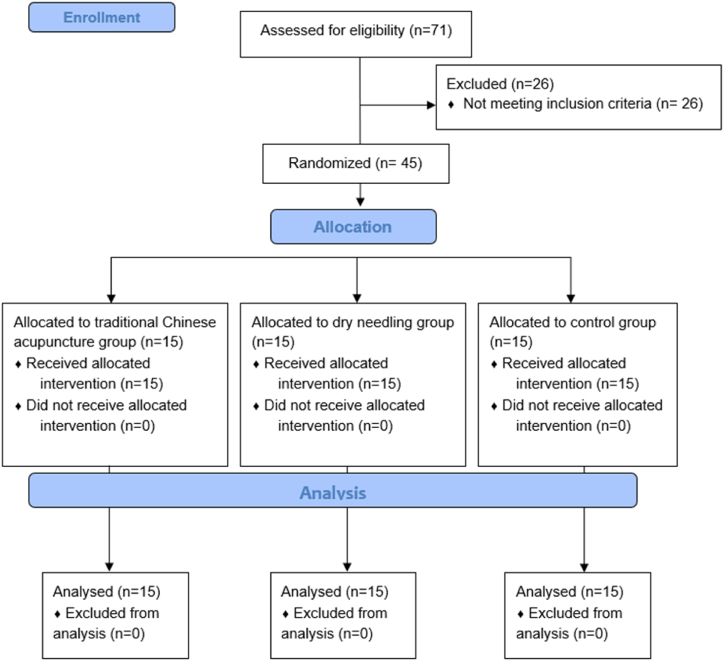
CONSORT flowchart.

A total of 71 volunteers were accounted to be eligible, although due to not reaching the eligibility criteria, 26 volunteers did not participate in the study. A total of 45 volunteers were divided into three groups: TCAg, DNg, and Cg (Fig. 3). Sample characterization and baseline values are presented in Table 1. According to IPAQ short version, 20 volunteers were considered “sedentary”, 13 as “irregularly active A” and 12 as “irregularly active B”.

**Table 1:** Sample characteristics and reference values (n = 45).

Characteristics	TCAg (mean ± SD) N = 15	DNg (mean ± SD) N = 15	Cg (mean ± SD) N = 15	*p*^a^
EMG (μV^)^	76.16 ± 10.01	78.15 ± 8.46	75.45 ± 7.41	0.68
ITI (°C)	31.93 ± 0.96	31.63 ± 0.75	31.50 ± 0.66	0.35
VAS	1.03 ± 1.10	1.60 ± 1.24	1.33 ± 1.54	0.50
Exercise time (seconds)	142.00 ± 68.05	116.00 ± 28.73	145.00 ± 64.90	0.31

TCAg: Chinese traditional acupuncture group; DNg: dry needling group; Cg: control group; EMG: EMG: electromyography; ITI: infrared thermographic imaging; VAS: visual analog scale; SD: standard deviation. ^a^ Reference values presented in the first measures of outcome variables (baseline); ^a^ ANOVA One Way test.

**Fig. 4:**
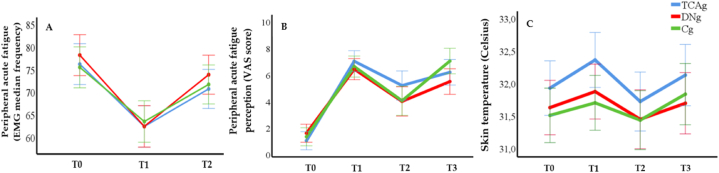
Results for muscle fatigue assessed with electromyography (EMG; A), muscle fatigue accessed with visual analog scale (VAS; B), and skin temperature (C).

Our results of electromyography indicate that all volunteers were successfully induced in a peripheral fatigue state of their biceps brachii, as stated in Fig. 4 (A), in which all volunteers presented a significant reduction of the MF (*p* ≤ 0.05) after not being able to reach at least 50%, demonstrating a fatigued state according to a decrease in the MF at T1, however, with no difference between groups. In addition, we express values comparing the T1 and T2 to indicate the treatment's effect, showing a significant increase in MF after treatment for all groups (*p* ≤ 0.05), however, with no difference between groups (*p=*0.824).

Fatigue perception analysis in Fig. 4 (B) revealed that the fatigue induction protocol was sufficient for the volunteer to experience peripheral fatigue in the biceps brachii (T1), and recover from this state after treatment (T2) (p ≤ 0.05), but there was no difference between analysis immediately after each fatigue induction (T1 vs. T3). In addition, intergroup analysis indicates a non-significant response to each moment comparison (*p* = 0.518).

We are presenting skin temperature results in Fig. 4 (C) showing both fatigue induction and treatment effects. All groups successfully obtained a significant result both for the temperature increase in fatigue induction (T1 and T3) and its decrease after the treatments (T2) (p ≤ 0.05). Therefore, comparing the two fatigue induction moments (T1 and T3), there was no difference between our results show no difference (*p* = 0.93). Likewise, the intergroup analysis revealed no significance between groups (*p* = 0.31).

**Fig. 5:**
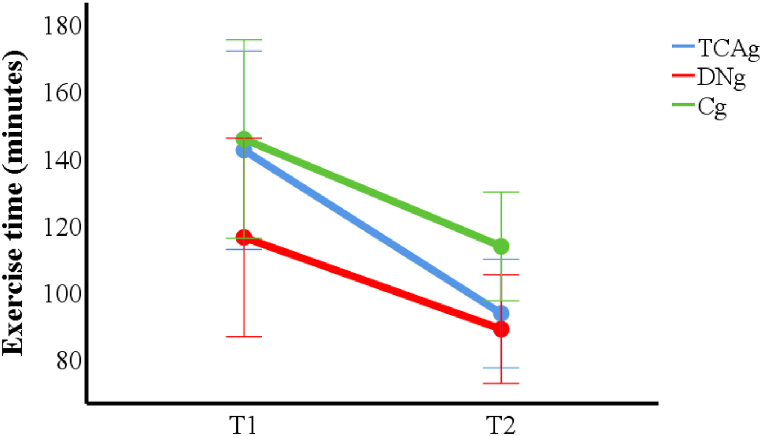
Results for exercise time for each fatigue induction.

We found that exercise time was reduced in the 2nd fatigue induction (T2) for all groups (p < 0.001), however, there is no difference among the groups (*p* = 0.21), indicating that the treatments did not interfere with the exercise time immediately after therapies (Fig. 5).

## 4 Discussion

We carry out this study to assess the immediate effects of TCA and DN on induced peripheral muscle fatigue over the biceps brachii of healthy and untrained volunteers. We hypothesized that it might reduce PAF, alter skin temperature, and exercise time in volunteers that underwent TCA would present better responses in their thermoregulatory system demonstrated by the vasoactive effects of TCA and DN. Our findings indicate that there are no differences between groups. However, we can state that our protocol effectively induces fatigue based on the changes observed in the median frequency (MF) of the EMG signals [2,53], and alters biceps brachii temperature both at fatigue induction and needling treatments as well as muscle fatigue perception.

Muscle contraction raises skin temperature due to increased blood flow, muscle tear, and inflammation secondary to a metabolic response proportional to the exercise, generating heat loss to the environment via convection and evaporation [54] permitting thermographic imaging to assess skin temperature. Therefore, skin temperature is related to muscle effort [55] as well as to muscle fatigue [17], indicating that a biceps brachii effort of an isometric exercise is going to generate peripheral fatigue with an alteration of skin temperature. Our study demonstrates the capability of a protocol [46] to generate peripheral muscle fatigue according to electromyographic, thermographic, and fatigue perception, according to Wolfe [56] and Kakuda et al. [38], it is possible to use VAS as an instrument for the perceived subject assessment of PAF.

The acupoints LI1, TH5, LI10, LI11, LI14, and GB21 exhibit the most significant influence on upper limb temperature changes [49] and we used a fast in and fast out technique was used for stimulation as described by Hong [57] and similarly used by Ershad et al., 2019 [25] for immediate effects. Studies indicate an increase in temperature immediately after needling insertion on DN [58,59]. However, our study identified a decrease in temperature after both TCA and DN. We hypothesize that the temperature decreased after the needle insertion of TCA and DN due to peripheral and systemic vasoconstriction initiated through an autonomic response, especially by sympathetic nerves [5], possibly indicating that the volunteers that experienced rest did not present a faster decrease in temperature due to not underwent TCA or DN.

Nevertheless, our study shows that there is an equal increase in temperature after the second fatigue induction. Volunteers were over the effects of vasoconstriction from TCA or DN before the fatigue induction protocol [5], however, due to a great effort to contract muscle fibers in isometric exercise, increased blood flow [55] overlapped needling effects. Since temperature increased equally for either technique, no better therapy induces a positive effect on skin temperature.

It is established that TCA treats muscle fatigue, possibly due to its nerve stimulation, known as “Deqi”, acting on myelinated and unmyelinated nerve fibers [60] increasing the recruitment of motor units and inducing the nervous system to increase muscle activity and pain processing matrix in the brain resulting in a descending pathway of serotonergic and noradrenergic liberation of endogenous opioids; thus muscle strength and pain relief should enhance physical performances [61]. We should also expect a similar physiological behavior regarding DN treatment [18] given that there are similarities in both techniques. Nonetheless, we found that TCN. DN and rest induce a positive effect over fatigue perception for induction and recovery equally to either treatment due to these effects.

A study proposes a correlation between acupoints and non-acupoints indicating the same neural mechanisms and autonomic phenomena including vasoconstriction [62]. Our study identifies a similarity between temperature and fatigue perception between TCA and DN indicating that both present the same behavior regarding thermoregulation reactions, which leads us to interpret as either therapy may work on muscle fatigue, especially since our results for fatigue perception are also aligned for both therapies as seen in previous studies [63,64].

These results are consistent with studies that evaluated the immediate effects of TCA [43,65] on exercise performance and muscular endurance, which did not find any difference in exercise time after the application of the treatment. To the best of our knowledge, there were no studies that assessed the acute effect of DN on muscular endurance, therefore, we might be inclined to rationale according to TCA, since it is also a needling treatment. However, there are indications that, in the medium and long term, there are positive effects on performance and endurance secondary to the application of TCA [18,19,66], and DN [67] as they change body physiology, different from the immediate effects that were presented in this study. Therefore, we believe that our results add that TCA and DN might be better to reduce skin temperature, however, we still need to determine what metabolic implications are inherent to these treatments and if skin reduction in temperature is indeed related to the decrease of PAF.

Therefore, further research is required to assess the mid to long-term effects of TCA and DN given that our study focuses on the acute effects of either treatment. Also, future studies should measure needling effects in disease-related populations that suffer from acute fatigue and an athlete-based population to better understand fatigue responses over TCA and DN over specific exercises. In addition, it is important to consider the unique methodology used in this study for further research concerning blinding and fatigue induction protocol.

## 5 Conclusions

In conclusion, the results of this investigation show that a sample of untrained healthy volunteers with induced PAF on their biceps brachii that overwent a protocol of TCA or DN presents no difference in PAF reduction, skin temperature, and exercise time. However, we believe that this behavior might be due to our small sample data and assessment only of acute effects.

Interestingly, intragroup analysis shows similar responses in each group, indicating that TCA, DN, and a resting state are effective, but do not differ. However, skin temperature shows a difference only for TCA and DN with a tendency towards intergroup significance, which might lead us to believe that the skin temperature of untrained healthy volunteers’ biceps brachii might be responsive to TCA and DN.

## Author contributions

Antonino G - Conceived and designed the experiments; Performed the experiments; Analyzed and interpreted the data; Wrote the paper.

Ferreira AP - Conceived and designed the experiments; Contributed reagents, materials, analysis tools or data.

Mendonça H - Performed the experiments.

Shirahige L - Contributed reagents, materials, analysis tools or data; Analyzed and interpreted the data.

Montenegro E − Conceived and designed the experiments; Contributed reagents, materials, analysis tools or data.

Guerino M − Contributed reagents, materials, analysis tools or data.

Filho A- Conceived and designed the experiments; Contributed reagents, materials, analysis tools or data.

Bernardo-Filho B - Conceived and designed the experiments; Contributed reagents, materials, analysis tools or data.

Campos SL - Contributed reagents, materials, analysis tools or data; Analyzed and interpreted the data.

Leite WS - Contributed reagents, materials, analysis tools or data; Analyzed and interpreted the data.

Monte-Silva K - Conceived and designed the experiments; Contributed reagents, materials, analysis tools or data; Analyzed and interpreted the data.

Taiar R - Analyzed and interpreted the data; Contributed reagents, materials, analysis tools or data; Wrote the paper.

Rapin A - Analyzed and interpreted the data; Contributed reagents, materials, analysis tools or data;

Araújo MGR - Conceived and designed the experiments; Contributed reagents, materials, analysis tools or data.

## Ethics statement

This study was approved by the Research Ethics Committee in Human Beings of the *Universidade Federal de Pernambuco* (Federal University of Pernambuco) under no. 2.419.094. All volunteers signed an informed consent form before the start of the study.

## Funding

This research was funded by Fundação de Amparo à Ciência e Tecnologia do Estado de Pernambuco (10.13039/501100006162FACEPE) APQ No. 0337–13. With thanks to Coordenação de Aperfeiçoamento de Pessoal de Nível Superior (CAPES).

## Informed consent statement

Informed consent was obtained from all subjects involved in the study.

## Declaration of competing interest

The authors declare that they have no known competing financial interests or personal relationships that could have appeared to influence the work reported in this paper.
